# Current therapeutic strategies of heterotopic ossification – a survey amongst orthopaedic and trauma departments in Germany

**DOI:** 10.1186/s12891-015-0764-2

**Published:** 2015-10-22

**Authors:** Sebastian Winkler, Ferdinand Wagner, Markus Weber, Jan Matussek, Benjamin Craiovan, Guido Heers, Hans Robert Springorum, Joachim Grifka, Tobias Renkawitz

**Affiliations:** Department of Orthopaedic Surgery, Regensburg University Medical Centre, Kaiser-Karl-V-Allee 3, 93077 Bad Abbach, Germany

**Keywords:** Heterotopic ossification, Trauma, Therapy, Fracture, Prophylaxis

## Abstract

**Background:**

Heterotopic ossification (HO) is a complication after tissue trauma, fracture and surgery (i.e. total hip arthroplasty). Prophylaxis is the most effective therapy. If HO formations become symptomatic and limit patients’ quality of life, revision surgery is indicated and is usually combined with a perioperative oral prophylaxis (NSAIDs) and/or irradiation. However, a long-term use of NSAIDs can induce gastro-intestinal or cardiac side-effects and possible bony non-unions during fracture healing. Subject of this study was to assess the current status of HO prophylaxis after injuries or fractures and to evaluate current indications and strategies for excision of symptomatic HO.

**Methods:**

Between 2013 and 2014, a questionnaire was sent to 119 orthopaedic and trauma surgery departments in Germany. Participation was voluntary and all acquired data was given anonymously.

**Results:**

The cumulative feedback rate was 71 %. Trauma and orthopaedic surgery departments in Germany recommend oral HO prophylaxis after acetabulum and femoral neck fractures, elbow dislocation, and fracture or dislocation of the radial head. Pain upon movement and an increasing loss of range of motion in the affected joint are considered to be clear indications for HO surgery. A partial removal of ROM-limiting HO formations was also considered important. The vast majority of all departments include perioperative oral HO prophylaxis and/or irradiation if surgical HO removal is planned. The choice and duration of NSAIDs is highly variable.

**Conclusion:**

HO is of clinical significance in current traumatology and orthopaedics. Certain fractures and injuries are prone to HO, and prophylactic measures should be taken. The respondents in this survey assessed current therapeutic strategies for HO formations similarly. These concepts are in line with the literature. However, the duration of perioperative oral HO prophylaxis varied greatly among the specialist centres. This is significant as a long-term use of NSAIDs fosters a potential risk for the patients’ safety and could influence the clinical outcome. National and international guidelines need to be developed to further reduce HO rates and improve patients’ safety in trauma and orthopaedic surgery.

## Background

Heterotopic ossification (HO) can occur after tissue trauma (i.e. gunshot wounds), cerebral and spinal cord injury, bone fractures or surgery such as total hip arthroplasty (THA) [1, 2]. The pathogenesis of HO is not fully understood.

An effective HO prophylaxis requires identifying known risk factors in the patient, using gentle surgical techniques and applying perioperative non-steroidal anti rheumatic drugs (NSAIDs), COX-2 blockers and low-dose irradiation [2]. This is well established for THA. Little is known about the significance of HO prophylaxis in blunt extremity trauma and fracture treatment. Nevertheless, some studies have suggested that NSAIDs, which suppress inflammatory prostaglandins during initial tissue inflammation, could cause delayed fracture healing in animals [3, 4]. Large and symptomatic HO formations can only be treated surgically [5]. However, revision surgery itself can induce a HO relapse.

Historically, Germany had a separation of trauma and orthopaedic surgery departments. Consequently, different therapy strategies evolved in both fields. Several years ago this separation was, however, revoked and a single residency program was created. Nonetheless, differences still remain in both disciplines.

The intention of this study was to investigate the following questions:

1. Is HO clinically relevant?
2. Are HO-prone injuries assessed in a similar manner in different specialist centres?
3. Are there standard HO prophylactic measures after fractures and tissue trauma?
4. What are the current indications and strategies for surgical HO removal if HO formations limit patients’ quality of life?

## Methods

After a current literature review, the authors developed a questionnaire. The 17 questions aimed to assess prophylactic and therapeutic strategies for heterotopic ossification in orthopaedic and trauma surgery. Indications for surgery and surgical techniques were graded on a 4-point scale (insignificant, of little importance, important or very important).

The study was carried out from 2013 to 2014 in 34 orthopaedic and 30 trauma surgery departments in German university hospitals. When there were separate departments in the same hospital, both departments were addressed. Furthermore, we contacted 55 German hospitals that had been granted a certificate (EndoCert®) for high standards in THA from the German Association of Orthopaedic Surgery and were listed on the official homepage until January 9th 2014. The study participants were trained orthopaedic/trauma surgeons, not patients. The respondents agreed to participate voluntarily in this trial by sending back the completed questionnaire in an anonymous fashion. Therefore, an informed consent was waived. The collected data included estimations, therapy assessments or ratings and did not allow any conclusion about the participants’ identity. An approval by the local IRB was waived according to the guideline for Good Clinical Practice and the Declaration of Helsinki as this trial did not involve the collection of sensitive data from humans.

### Statistical analysis

Statistics were generated with Microsoft Excel 2008. Statistics used for this study were descriptive only as several answers to the questions included estimations from the questionnaire recipients. Hence, standard parameters such as mean, median, maximum and minimum were detected, but no statistical analysis for significance was performed.

## Results

The total response rate was 71 % (85 of 119). The feedback rate was 76 % (42 of 55) among non-university hospitals, 67 % (20 of 30) in trauma and 68 % (23 of 34) in the orthopaedic departments of university hospitals.

The majority of surgeons offer a regular radiological follow-up that enables a realistic assessment of the course of treatment. Most respondents assessed HO-prone injuries in a similar manner and therefore recommend an oral HO prophylaxis after certain fractures (see Table 1 for details).

**Table 1:** HO prophylaxis in fracture treatment and summary of injuries and fracture types that are prone to HO formation according to respondents; HO = heterotopic ossification; AC = acromio-clavicular; min. = minimum, max. =maximum

		Germany		
	University (trauma)	University (orthopaedic)	Non-university hospitals	Total
Feed back rate	67 % (*n* = 20 of 30)	68 % (*n* = 23 of 34)	76 % (*n* = 42 of 55)	71 % (*n* = 85 of 119)
HO prophylaxis for risk fractures	79 %	67 %	59 %	67 %
Mean time of prophylaxis (days)	25 (min. 14 – max. 42)	17 (min. 7 – max. 42)	21 (min.7 – max.84)	21 (min. 7 – max. 84)
Regular radiological follow-up	85 %	96 %	67 %	79 %
Injuries and fractures prone to HO:				
Acetabulum fracture	79 %	52 %	38 %	54 %
Elbow dislocation	42 %	24 %	31 %	32 %
Radial head dislocation	16 %	5 %	10 %	10 %
Radial head fracture	21 %	14 %	10 %	14 %
Femoral neck fracture	11 %	48 %	31 %	30 %
Femoral shaft fracture	0 %	24 %	17 %	14 %
Clavicula fracture	0 %	5 %	7 %	4 %
AC joint injury	0 %	5 %	7 %	4 %
Other	0 %	0 %	0 %	0 %

The indications for HO excision were assessed similarly. Pain at rest, pain during joint movement and a reduced range of motion (ROM) are considered important or very important. Thirty-seven percent of the respondents found a skeleton scintigraphy to be useful. The evaluation of surgical strategy and techniques for HO excision were moderately different among orthopaedic and trauma surgeons (see Table 2 for details).

**Table 2:** Indication and techniques for surgical HO removal; HO = heterotopic ossification

		Germany		
	University (trauma)	University (orthopaedic)	Non-university hospitals	Total
Indications for surgery				
Pain at rest				
Insignificant	10 %	0 %	0 %	3 %
Of little importance	40 %	17 %	22 %	25 %
Important	35 %	57 %	67 %	55 %
Very important	15 %	26 %	11 %	17 %
Pain during joint movement				
Insignificant	0 %	0 %	0 %	0 %
Of little importance	11 %	0 %	0 %	3 %
Important	53 %	64 %	69 %	64 %
Very important	37 %	36 %	31 %	33 %
Reduced ROM of affected joint				
Insignificant	0 %	0 %	0 %	0 %
Of little importance	5 %	9 %	3 %	5 %
Important	20 %	43 %	56 %	43 %
Very important	75 %	48 %	41 %	52 %
Increase of HO formation				
Insignificant	16 %	13 %	14 %	14 %
Of little importance	26 %	39 %	36 %	34 %
Important	42 %	44 %	28 %	37 %
Very important	16 %	4 %	22 %	14 %
Active HO formation in scintigraphy				
Insignificant	21 %	4 %	14 %	13 %
Of little importance	63 %	61 %	39 %	50 %
Important	16 %	26 %	33 %	28 %
Very important	0 %	9 %	14 %	9 %
Techniques of surgical excision				
Complete excision of HO formation				
Insignificant	5 %	0 %	3 %	3 %
Of little importance	40 %	36 %	30 %	34 %
Important	45 %	55 %	53 %	51 %
Very important	10 %	9 %	14 %	12 %
Excision of ROM-limiting HO				
Insignificant	5 %	0 %	2 %	3 %
Of little importance	15 %	30 %	23 %	22 %
Important	30 %	48 %	46 %	43 %
Very important	50 %	22 %	29 %	32 %
Tissue interposition after HO removal				
Insignificant	45 %	13 %	25 %	27 %
Of little importance	40 %	70 %	61 %	60 %
Important	10 %	17 %	14 %	13 %
Very important	5 %	0 %	0 %	0 %

In HO excision, the majority of surgeons change their HO prophylactic strategy by applying irradiation and/or administering a different NSAID. The choice of medication and the amount of time it was taken were inconsistent. Irradiation is usually planned pre-operatively in Germany (see Table 3 for details).

**Table 3:** Perioperative irradiation and oral HO prophylaxis; HO = heterotopic ossification; Gy = Gray

		Germany		
	University (trauma)	University (orthopaedic)	Non-university hospitals	Total
Postoperative oral HO prophylaxis	100 %	81 %	86 %	88 %
Perioperative irradiation	90 %	96 %	91 %	92 %
Change in prophylaxis strategy	65 %	35 %	31 %	38 %
Diclofenac	0 %	25 %	30 %	17 %
*Median dosage per day*	-	150 mg	150 mg	150 mg
Ibuprofen	11 %	25 %	10 %	13 %
*Median dosage per day*	1800 mg	1200 mg	1200 mg	1600 mg
Indomethacin	89 %	50 %	30 %	57 %
*Median dosage per day*	150 mg	150 mg	100 mg	100 mg
Etoricoxib	0 %	-	20 %	9 %
*Median dosage per day*	-	-	90 mg	90 mg
Celecoxib	0 %	-	10 %	4 %
*Median dosage per day*	-	-	400 mg	400 mg
Other	-		-	0 %
Mean time of oral prophylaxis (days)	28 (min.14 – max.42)	42 (min.14 – max.98)	21 (min.7 – max.42)	30 (min.7 – max.98)
Pre-operative irradiation	75 %	86 %	90 %	83 %
Post-operative irradiation	25 %	14 %	10 %	17 %
Times of irradiation	1	1	1	1
Median dosage in Gy	7	7	7	7

## Discussion

In recent years, strategies for HO in total hip arthroplasty have been discussed in various studies [6]. A national treatment guideline in Germany (2009) recommends NSAID use for HO prophylaxis after THA and elbow injuries/surgery [7]. No recommendation was given for fractures, tissue trauma and spinal or cerebral injuries. Fracture osteosynthesis and manipulation of soft tissue and joints seem to be HO risk factors [8, 9]. Gunshot or blast injuries can also cause HO formations [10].

The aim of this study was to evaluate current prophylactic strategies after injuries and to analyse therapeutic concepts of symptomatic HO. Additionally, we intended to investigate whether data taken from recent literature had found its way into daily trauma and orthopaedic practice. However, this review has certain limitations. Surveys usually cannot provide strong evidence of cause and effect. Future studies could include face to face follow-up visits for patients who were treated for HO, since the subjective error from patients is hard to remove. Secondly, the survey included closed questions, which are generally considered to be of lesser validity than open-ended questions, which were also included. Ultimately, the results of this study are limited by response rate and the subjective estimations from the respondents. The response rate was 71 % (85/119), which is considered high [11, 12].

A main finding of this study is that orthopaedic and trauma surgeons alike consider HO to be clinically relevant after certain fractures and injuries. Nevertheless, there are differences in current HO prophylaxis. Trauma surgery departments have a higher rate of HO prophylaxis after risk fractures/injuries compared to orthopaedic university departments and non-university EndoCert® hospitals. The oral HO prophylaxis duration was also slightly higher among trauma surgeons, but on average for three weeks post trauma.

The literature provides numerous clinical trials which found that an oral HO prophylaxis (indomethacin) can reduce the incidence of large HO formations (Brooker grades 3 and 4) after certain fractures [13]. It must be taken into account that NSAIDs can lead to delayed fracture healing and non-unions in animals [3, 4]. Despite these results, many patients receive NSAIDs for both, postoperative pain and anti-inflammatory (HO prophylactic) treatment after trauma. An evidence- based recommendation for the most effective drug and perioperative application time for oral HO prophylaxis is currently missing from the literature.

The respondents in this study mostly considered acetabular fractures, elbow dislocation, femoral neck and shaft fractures and radial head dislocation or fracture to be most prone to HO. Current literature supports this assessment. A recent retrospective study (2013) found a 47 % incidence of HO (56 of 120 patients) after surgery for acetabular fracture [14]. After elbow fracture surgery involving the proximal radius and/or ulna, HO was reported in 37 % of cases (48 of 142) [15]. Johansson et al. analysed femoral neck fracture treatment (THA vs. internal fixation): Seventy-one percent (32 of 45) of hips with THA developed HO compared to 2.5 % (1 of 39) in the internal fixation group [16]. Mechanical ventilation time, indomethacin and incidence of head injuries did not differ between the two groups. Plate osteosynthesis seems to create a higher risk for HO formation than intramedullary nailing in multiple trauma patients [17]. A possible explanation might be that plate osteosynthesis is often used for intraarticular fracture treatment and could induce higher muscle injuries, whereas intramedullary nailing is mostly used for diaphysal fractures. Another risk factor for HO formation in multiple trauma patients is brain injury. A higher rate of HO is found when serious brain injury occurred and there was a need for mechanical ventilation [18].

If HO formations increase, they can cause postoperative pain, bony impingement with impaired range of motion of the affected joints, leading ultimately to revision surgery. This survey shows that current indications and strategies for surgical HO removal are similar with trauma and orthopaedic surgeons. Surgical removal of HO is complicated as the formations are often soft and integrated in the muscle or connective tissue. Some studies demonstrated that excision of HO can lead to a significantly improved ROM, but pain can often not be minimized [19]. Salazar et al. [20] described a significant, postoperative improvement in ROM after resection of HOs following elbow fractures when preoperative ROM was partially or completely restricted. Consequently surgeons should carefully consider these results from the literature when HO excision is indicated. At this stage, there is limited data as to whether HO formations should be removed completely or partially.

If revision surgery is indicated, the vast majority of surgeons combine irradiation and an oral prophylaxis in their therapy regimen. However, this review has found that there are still vast differences in the choice of drug and the perioperative application time (minimum 7 days, maximum 98 days). A majority of trauma surgeons prefer the use of indomethacin in HO surgery.

An evidence-based recommendation for the most effective HO prophylaxis (type of drug, application time) following HO excision is still missing in the current literature. The combination of irradiation and NSAID use for HO treatment of various joints has been successfully described [21]. The outcome of irradiation does not seem to increase the risk of malignancy [22].

There are few alternative or new treatment options for HO: The long-term effect of bisphosphonates to prevent HO remains unclear [23]. Basic research found selective retinoic acid receptor γ (RAR γ) agonists able to suppress BMP signalling and chondrogenesis in a murine model [24]. Inhibitors of substance P and mast cell blockers (cromolyn) showed significant reduction in HO rates in animals and seem to be promising future therapeutics [25]. The use of RNAi might become an additional new alternative [26]. These options are currently at an experimental stage but could become available in the near future to further reduce HO rates in orthopaedic and trauma surgery.

## Conclusion

This survey indicates that orthopaedic and trauma surgeons in Germany consider HO to be of clinical relevance in their daily practice. The injuries that are at risk of HO and require HO prophylaxis were usually assessed in a similar manner. Indications for surgery of symptomatic HO formations and surgical techniques were equally weighted and are in concordance with the literature. The perioperative oral prophylaxis (i.e. choice of NSAID or COX-2 blocker, application duration) which is often combined with revision surgery was highly variable. This is significant for clinical practice as a long-term use of NSAIDs fosters a potential risk to the patients’ safety and could influence the clinical outcome. This survey was able to demonstrate that in 2014 there was still a need for clinical trials as well as national and international treatment guidelines to detect the most effective HO prophylaxis for risk fractures/injuries and following HO excision. This could further reduce HO rates and improve patients’ safety in trauma and orthopaedic surgery.

## Abbreviations

AC: Acromio-clavicular
BMP: Bone morphogenetic protein
COX: Cyclooxygenase
EndoCert®: A certificate for high standards in THA granted by the German Association of Orthopaedic Surgery
Gy: Gray
HO: Heterotopic ossification
min.: Minimum
max.: Maximum
NSAID: Non-steroidal anti-inflammatory drug
RAR γ: Retinoic acid receptor γ
RNAi: Ribonucleic acid i
ROM: Range of motion
THA: Total hip arthroplasty
